# GLP-1-Based therapies for obesity-related heart failure with preserved ejection fraction: an umbrella review incorporating broader heart failure and cardiometabolic evidence

**DOI:** 10.3389/fcvm.2026.1930278

**Published:** 2026-09-30

**Authors:** Qiang Li, Tianyi Yang, Guannan Jin, Yabin Zhou

**Affiliations:** 1 Heilongjiang University of Chinese Medicine, Harbin, Heilongjiang, China; 2 Heilongjiang Academy of Chinese Medicine Sciences, Harbin, Heilongjiang, China

**Keywords:** cardiometabolic therapy, GLP-1 receptor agonists, heart failure, HFpEF, obesity, semaglutide, tirzepatide, umbrella review

## Abstract

**Background:**

The cardiometabolic phenotype of obesity-related heart failure with preserved ejection fraction (HFpEF) is increasing in clinical prevalence. Existing systematic reviews and meta-analyses have primarily summarized efficacy estimates for glucagon-like peptide-1 (GLP-1)-based therapies, but the credibility and independence of these syntheses remain uncertain because methodological limitations and overlap in primary trials have not been systematically evaluated. This umbrella review therefore appraised the methodological quality, overlap, consistency, and robustness of the existing review-level evidence rather than generating another efficacy synthesis.

**Methods:**

We conducted an umbrella review of systematic reviews, meta-analyses, and network meta-analyses evaluating GLP-1-based regimens in obesity-related HFpEF and adjacent cardiometabolic populations. PubMed, Embase, Web of Science, and the Cochrane Library were searched over the preceding decade. The systematic review or meta-analysis was the unit of analysis. Methodological quality was assessed with AMSTAR 2, and overlap of primary studies across reviews was quantified using the corrected covered area (CCA). No *de novo* meta-analysis was performed; primary trials were examined only to assess overlap and calculate the CCA. Review-level findings were synthesized according to effect direction, consistency, precision, sample size, methodological quality, clinical relevance, and overlap.

**Results:**

Seventeen systematic reviews underwent qualitative profiling, of which 11 reported quantitative estimates relevant to the core review-level synthesis. For heart failure hospitalization, the review by Diallo et al. reported HR 0.78 (95% CI 0.73–0.84; *n* = 25,440). For the composite of cardiovascular death and heart failure hospitalization, Diallo et al. reported HR 0.84 (95% CI 0.78–0.89), Ahmed HM et al. reported HR 0.76 (95% CI 0.64–0.90), and Ahmed M et al. reported RR 0.73 (95% CI 0.57–0.93). Existing reviews also consistently suggested improvements in functional status. The review by Gera et al. reported OR 0.85 (95% CI 0.77–0.93; *n* = 85,373) for stroke, whereas findings for cardiovascular and all-cause mortality remained inconsistent. Evidence for major adverse cardiovascular events was limited; the review by Gonzales-Uribe et al. reported HR 0.71 (95% CI 0.60–0.86). AMSTAR 2 classified six reviews as having low overall methodological confidence and five as having critically low overall methodological confidence. The CCA was 13.2%, indicating high overlap. All HR, RR, and OR values were estimates reported by included reviews and were not recalculated or quantitatively combined in this umbrella review.

**Conclusions:**

At the level of existing evidence syntheses, GLP-1-based therapies appear most consistently associated with fewer heart failure hospitalizations and improved functional capacity in obesity-related HFpEF. However, the review-level evidence does not establish a consistent mortality benefit or support GLP-1-based therapy as a universal mortality-modifying treatment for HFpEF. These agents are better interpreted as emerging metabolic-functional therapies whose apparent benefits must be considered in light of methodological limitations and high overlap among reviews.

## Introduction

Driven predominantly by the dual burden of obesity and systemic metabolic dysregulation, heart failure with preserved ejection fraction (HFpEF) has emerged as a pervasive cardiometabolic disorder (1–3). Insights garnered over the last ten years have thoroughly overhauled our understanding of HFpEF, transitioning it from a simplistic anomaly of preserved systolic mechanics into a complex, systemic, and highly heterogeneous syndrome. Within this modernized paradigm, systemic inflammatory cascades, microvascular impairment, metabolic disturbances, and pathological myocardial remodeling are recognized as central pathogenic drivers (4–7). Parallel to this mechanistic shift, glucagon-like peptide-1 (GLP-1)-based regimens—initially pioneered for glycemic optimization—have undergone a profound clinical repositioning as potent cardiometabolic therapeutics. Large-scale randomized trials have consistently validated their efficacy in facilitating weight loss and ameliorating cardiovascular risk profiles across diverse cardiometabolic cohorts (8–17). This therapeutic momentum has been further catalyzed by recent landmark outcome trials—most notably SELECT, STEP-HFpEF, STEP-HFpEF DM, SUMMIT, and FLOW—which underscore the tangible clinical value of these agents in improving both functional milestones and circulatory outcomes in targeted populations (18–22). Nonetheless, the clinical impact of GLP-1 therapies within the specific realm of HFpEF remains notably discordant across divergent endpoints.

Numerous systematic reviews and meta-analyses have summarized this expanding evidence base, generally reporting fewer heart failure-related admissions and improvements in exercise tolerance and health-related quality of life (23–33). Their conclusions diverge, however, for cardiovascular mortality and major adverse cardiovascular events (MACE). Most previous syntheses focused on estimating treatment efficacy within selected populations and outcomes; they did not jointly evaluate the methodological credibility of the reviews, the independence of their underlying trial evidence, or the robustness of agreement across reviews. Differences in patient characteristics, intervention definitions, and endpoint selection are compounded by repeated inclusion of the same landmark primary trials, leaving the independent evidentiary weight of the published syntheses uncertain (34–37).

Accordingly, the purpose of this umbrella review was not to produce another treatment-effect estimate. Its novelty lies in evaluating the credibility of the existing evidence synthesis: we assessed the methodological quality of reviews using AMSTAR 2, quantified overlap among reviews using the corrected covered area (CCA), and examined the consistency and robustness of review-level conclusions across cardiovascular, heart failure, and functional outcomes. By integrating methodological quality, clinical relevance, and the extent of shared primary-trial evidence, this approach sought to distinguish findings supported by relatively robust review-level evidence from apparent agreement that may be driven by duplicated evidence.

## Methods

### Study design

This umbrella review consolidated systematic reviews, meta-analyses, and network meta-analyses that evaluated GLP-1-based regimens in populations with obesity-related HFpEF or adjacent cardiometabolic conditions. The approach was selected because, despite the proliferation of secondary evidence, no prior synthesis had jointly assessed methodological quality, review overlap, consistency of review-level evidence, and directional concordance. The systematic review or meta-analysis was the unit of analysis. Individual primary trials were examined only to map cross-review overlap and calculate the CCA; they were not treated as independent units for outcome synthesis, and their effect estimates were not quantitatively recombined.

The review followed established methodological guidance for umbrella reviews (34) and was reported in accordance with PRISMA 2020 (38). The analysis remained at the review level and did not deconstruct or re-aggregate data from individual primary studies. Its purpose was to map areas of agreement and uncertainty, assess whether published conclusions were supported by sound review methods, and determine the extent to which apparently independent reviews relied on shared primary evidence. This umbrella review was not prospectively registered in PROSPERO.

### Literature search strategy

To identify potentially eligible systematic reviews, meta-analyses, and network meta-analyses focused on GLP-1-based regimens within HFpEF and allied cardiometabolic cohorts, a comprehensive, systematic literature retrieval was executed. The electronic databases thoroughly queried encompassed PubMed, Embase, Web of Science, and the Cochrane Library. The search window was temporally restricted to the preceding ten years, culminating on June 30, 2026. The search architecture harmonized controlled vocabulary paradigms (such as Medical Subject Headings) with an exhaustive array of free-text keywords capturing the core domains of population, intervention, and study design. Specifically, key search strings targeted heart failure with preserved ejection fraction (HFpEF), heart failure with mildly reduced ejection fraction (HFmrEF), obesity, cardiometabolic syndromes, specific GLP-1 receptor agonists and co-agonists (including semaglutide, liraglutide, exenatide, and tirzepatide), alongside secondary evidence synthesis methodologies.

Boolean operators were adapted to the syntax of each database to balance sensitivity and specificity. Reference lists of candidate reviews and authoritative articles were screened manually to identify potentially eligible reports not captured electronically. In keeping with the umbrella review design, no *de novo* meta-analysis of primary-study or participant-level data was undertaken; the analysis was restricted to appraisal and synthesis of published review-level evidence. Complete database-specific search strategies, including field tags and limits, are provided in Supplementary Table S3.

### Eligibility criteria

Adhering to a prespecified PICO framework, eligibility benchmarks were established *a priori*. Eligible clinical cohorts comprised adults presenting with phenotypic HFpEF, obesity-driven HFpEF, or HFmrEF, alongside adjacent cardiometabolic populations, on the condition that heart failure-related sequelae were explicitly cataloged. Concurrently, secondary studies capturing individuals burdened by type 2 diabetes, overweight/obesity, cardiovascular risk determinants, or established macrovascular pathologies were deemed acceptable, provided they rendered applicable data concerning obesity-related HFpEF endpoints.

The interventional exposure under evaluation was restricted to GLP-1-based regimens, embracing both selective GLP-1 receptor agonists (such as semaglutide, liraglutide, and exenatide) and twin GLP-1/GIP receptor co-agonists (such as tirzepatide). These were weighed against control arms consisting of placebos, conventional standard of care, or active control variants as structured within the original review protocols. The target clinical endpoints spanned cardiovascular-specific mortality, heart failure hospitalizations, composite circulatory events (cardiovascular death aggregated with heart failure admission or clinical deterioration), all-cause mortality, major adverse cardiovascular events (MACE), and cerebrovascular stroke. Additionally, patient-reported and functional outcomes were gauged via health-related quality of life metrics (specifically KCCQ) and functional exercise capacity (indexed by 6MWD).

Methodologically, eligible architectures were limited to systematic reviews, pairwise meta-analyses, and network meta-analyses (36). Conversely, narrative synthesis reviews, editorial commentaries, expert perspective pieces, conference abstracts, academic posters, redundant literature, and correspondence lacking raw quantitative datasets were systematically disqualified. In instances where multiple overlapping reviews targeted an identical clinical inquiry, the final choice for core data synthesis favored the artifact demonstrating the highest comprehensiveness and methodological integrity.

### Study selection process

All bibliographic citations captured through electronic execution were migrated to EndNote software to facilitate centralized reference stewardship. Duplicated citations were systematically purged leveraging an initial automated algorithm supplemented by painstaking manual verification. The subsequent vetting process strictly conformed to the PRISMA 2020 procedural roadmap.

The database search identified 1,745 records. After removal of 218 duplicates, 1,527 titles and abstracts were screened, and 35 reports underwent full-text assessment against the prespecified eligibility criteria. Eighteen reports were excluded for documented reasons, including non-target populations, ineligible interventions, absence of prespecified clinical outcomes, narrative review design, or abstract-only publication. Seventeen systematic reviews were included in the qualitative profile, and 11 contributed quantitative estimates reported by the reviews to the core review-level evidence synthesis and methodological appraisal. The selection process is shown in the PRISMA flow diagram.

### Data extraction

Data harvesting was executed utilizing a uniform repository template specifically engineered for the operational needs of this umbrella review. The matrices compiled from each eligible secondary study spanned three core dimensions: bibliographic attributes (comprising primary authorship, calendar year of publication, and country of origin), methodological metrics (encompassing the review design, quantity and baseline profile of encapsulated primary studies, alongside the aggregate cohort volume), and granular clinical specificities (covering detailed patient demographics, precise drug dosing/comparator architectures, specific endpoint parameters, relevant point estimates flanked by their 95% confidence intervals, statistical *p*-values, and the overarching thematic conclusions).

Quantitative measures, including hazard ratios (HRs), risk ratios (RRs), odds ratios (ORs), and mean differences (MDs), were transcribed as reported in the included reviews and assigned to prespecified outcome domains. These values were treated as review-reported estimates and were not recalculated or quantitatively recombined by this umbrella review. When multiple overlapping reviews addressed the same endpoint, interpretation prioritized methodological quality, sample size, population relevance, precision, and applicability to obesity-related HFpEF. Primary-trial information was used only to identify cross-review citation patterns and calculate the CCA. Missing items were recorded as “not reported” in the evidence matrix.

### Quality assessment

The methodological quality of the 11 reviews contributing to the core review-level evidence synthesis was assessed using A MeaSurement Tool to Assess Systematic Reviews 2 (AMSTAR 2) (35). AMSTAR 2 evaluates 16 domains spanning protocol development, literature searching, study selection, data extraction, risk-of-bias assessment, quantitative synthesis methods used by the included reviews, publication-bias assessment, and reporting transparency. Each domain was rated as “Yes,” “Partial Yes,” or “No” according to the instrument's guidance. Overall methodological confidence was categorized as high, moderate, low, or critically low according to the presence of critical and non-critical weaknesses. These categories refer exclusively to methodological confidence according to AMSTAR 2 and should not be interpreted as evidence certainty ratings.

Critical AMSTAR 2 domains included *a priori* protocol registration, search completeness, documentation of excluded studies, risk-of-bias assessment, appropriateness of quantitative synthesis methods used by the included reviews, consideration of risk of bias when interpreting results, and assessment of publication bias. These ratings were used to calibrate interpretation of review-level findings. They were not treated as direct ratings of evidence from individual primary trials.

### Citation overlap analysis

To map the degree of structural redundancy among the 11 core secondary investigations, a comprehensive cross-citation matrix was engineered. Within this analytical grid, distinct primary clinical trials populated the rows, while the encapsulated reviews served as the column vectors. To circumvent misclassification, individual primary publications were cross-referenced and standardized based on proprietary trial nomenclatures, study acronyms, and chronological publication tracking, ensuring unequivocal tracking of shared evidence paths.

The extent of overlap was quantified using the corrected covered area (CCA): CCA = (N − c)/(rc − c), where N is the total number of primary-study occurrences across reviews, r is the number of included reviews, and c is the number of unique primary studies (37). CCA values were interpreted according to established thresholds: 0%–5% as slight overlap, 6%–10% as moderate overlap, 11%–15% as high overlap, and greater than 15% as very high overlap. Pairwise overlap was also examined to characterize the distribution of shared primary evidence between individual reviews.

### Evidence synthesis

Evidence was synthesized at the review level using a structured narrative approach. No *de novo* meta-analysis was performed, and primary-trial estimates were not quantitatively recombined. Extracted findings were organized into prespecified domains covering cardiovascular events, heart failure outcomes, and functional status. Where network meta-analyses were available, their estimates were interpreted as reported by the original authors and considered alongside the assumptions and analytical pathways of those reviews, with reference to PRISMA-NMA guidance where applicable (36).

For each outcome, direction was first described as beneficial, neutral, harmful, or uncertain. Review-level findings were then assigned a prespecified umbrella-review interpretative classification. Consistent review-level evidence indicated a stable direction across the most relevant reviews; moderately consistent evidence indicated general directional agreement constrained by methodological limitations, imprecision, or overlap; limited consistency across reviews indicated sparse, heterogeneous, indirect, or population-limited evidence; and uncertain indicated no coherent directional pattern. Classification integrated AMSTAR 2 methodological confidence, consistency of direction, precision, sample size, clinical relevance, and the extent of primary-study overlap.

These umbrella-review interpretative classifications are not formal GRADE ratings, and no GRADE assessment was performed. When several reviews addressed the same endpoint, greater interpretive weight was given to reviews with stronger methods, more complete searches, more precise estimates, larger samples, and closer relevance to obesity-related HFpEF. Overlap was considered explicitly so that repeated analyses of the same primary trials were not interpreted as independent confirmations. Primary-trial results were not counted or quantitatively combined again; conclusions were based on the direction and robustness of review-level evidence.

## Results

### Literature search and selection

The database search identified 1,745 records. After removal of 218 duplicates, 1,527 records underwent title and abstract screening, and 1,492 were excluded. Thirty-five full-text reports were assessed for eligibility, of which 18 were excluded: weight-loss-only reviews (*n* = 5), narrative reviews (*n* = 4), conference abstracts or posters (*n* = 3), reviews reporting non-cardiovascular outcomes (*n* = 3), reviews of populations outside the prespecified scope (*n* = 2), and one duplicate publication (*n* = 1). Seventeen reviews were included in the qualitative synthesis, and 11 contributed reported quantitative estimates to the core review-level evidence synthesis. The selection process is presented in the PRISMA 2020 flow diagram (Figure 1).

**Figure 1:**
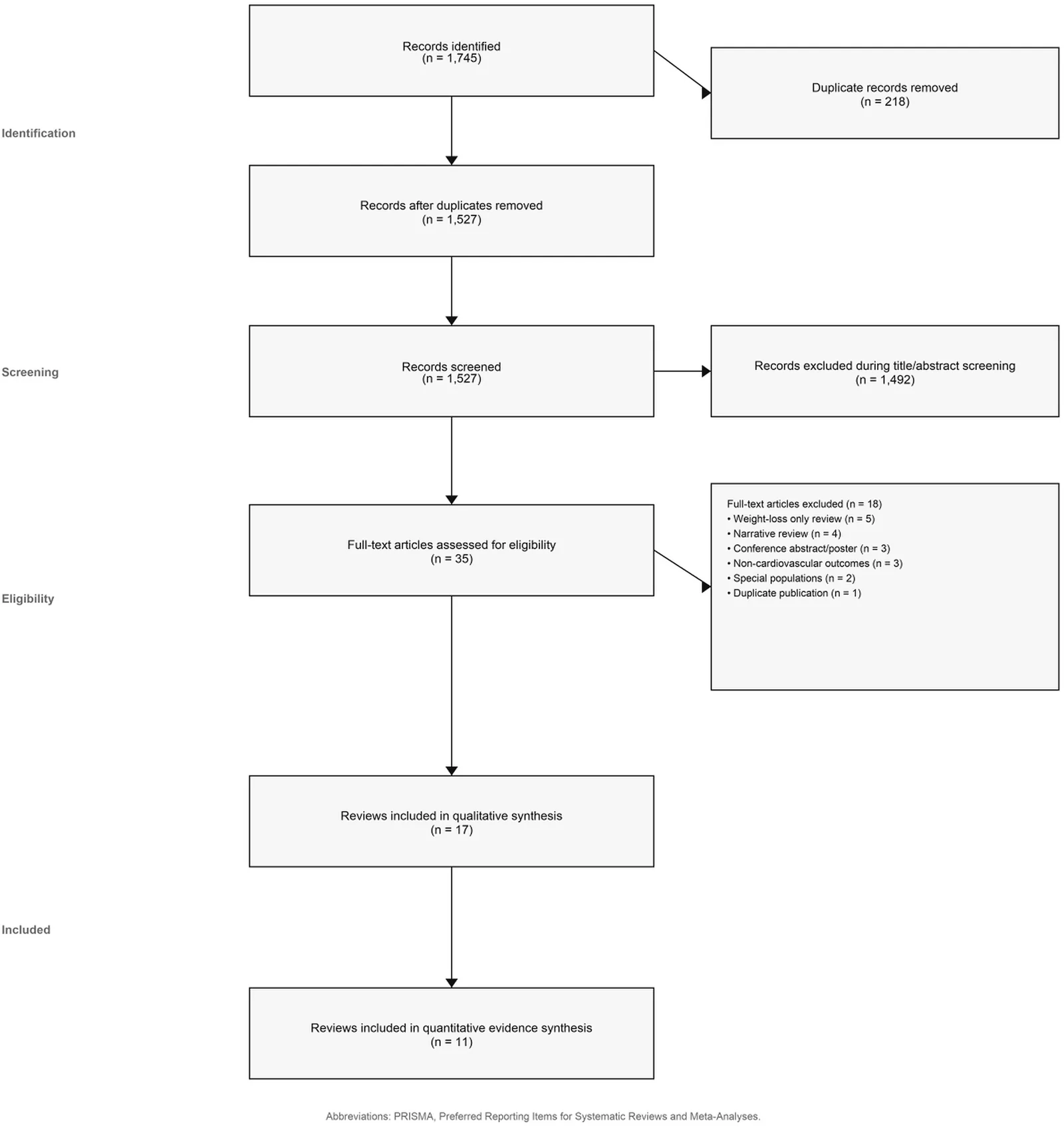
PRISMA 2020 flow diagram of review selection. The diagram shows records identified, duplicates removed, title and abstract screening, full-text assessment, reasons for full-text exclusion, reviews included in qualitative profiling, and reviews contributing reported quantitative estimates to the core review-level synthesis.

### Characteristics of included reviews

The 11 reviews retained for the core review-level evidence synthesis were published between 2022 and 2026, with most appearing in 2025 or 2026. As summarized in Table 1, they evaluated a broad range of populations, including HFpEF, obesity-related HFpEF, heart failure with mildly reduced ejection fraction (HFmrEF), selected heart failure with reduced ejection fraction subgroups, people with overweight or obesity with or without type 2 diabetes, and broader cardiovascular outcome-trial populations. Consequently, estimates from broader cardiometabolic cohorts were treated as supportive context rather than definitive evidence specific to obesity-related HFpEF. To improve interpretability, included reviews were therefore classified according to their evidence context, distinguishing HFpEF-specific evidence, heart failure-related evidence from mixed HF phenotypes, and cardiometabolic supportive evidence (Table 1). Five reviews were classified as HFpEF-specific evidence, three as heart failure-related evidence, and three as cardiometabolic supportive evidence. This classification distinguished direct phenotype-specific evidence from indirect contextual evidence throughout the synthesis.

**Table 1:** Characteristics of included systematic reviews, meta-analyses, and network meta-analyses.

Study (First author, Year)	Population	Evidence context	Intervention	Comparators	Sample size (range)	Key outcomes
Ahmed (23)	HFpEF/HFrEF	Heart failure-related evidence	GLP-1 RAs	Placebo/control	11,234	HF worsening, CV death, functional outcomes
Ahmed (24)	HFmrEF/HFpEF	Heart failure-related evidence	GLP-1/GIP agonists	Usual care	—	CV outcomes, QoL
Chen (28)	HFpEF	HFpEF-specific evidence	GLP-1 RAs	Control	—	Functional outcomes
Costa (30)	Obesity-related HFpEF	HFpEF-specific evidence	GLP-1 RAs	Placebo	—	6MWD, QoL
Diallo (31)	HFpEF/HFmrEF	Heart failure-related evidence	GLP-1 RAs	Comparator therapy	—	MACE, mortality
Gonzales-Uribe (33)	HFpEF	HFpEF-specific evidence	GLP-1 RAs	Control	—	CV outcomes
Patoulias (27)	Overweight/obesity	Cardiometabolic supportive evidence	Semaglutide	Placebo	—	CV safety
Cleto (29)	Obesity	Cardiometabolic supportive evidence	Semaglutide	Placebo	—	Stroke, CV outcomes
Caldeira Gaelzer (26)	Obesity-related HFpEF	HFpEF-specific evidence	GLP-1 RAs	Control	—	QoL, HF events
Behers (25)	HFpEF	HFpEF-specific evidence	GLP-1 RAs	Control	—	HF outcomes
Gera (32)	With/without diabetes	Cardiometabolic supportive evidence	GLP-1 RAs	Placebo/control	85,373	Stroke

In terms of pharmacological exposure, the investigated therapeutic strategies focused centrally on selective GLP-1 receptor agonists—predominantly semaglutide, liraglutide, and exenatide—alongside dual incretin co-agonists exemplified by tirzepatide. Intriguingly, several secondary syntheses conceptualized GLP-1-based regimens within expanded cardiometabolic therapeutic meshes, juxtaposing or pairing them with sodium-glucose cotransporter-2 (SGLT2) inhibitors, mineralocorticoid receptor antagonists (MRAs), angiotensin receptor-neprilysin inhibitors (ARNIs), and conventional heart failure therapeutics optimized for the HFpEF phenotype (28, 31, 39–43). The documented clinical endpoints formed a comprehensive grid, integrating macrovascular events, heart failure-related decompensations, mortality metrics, cerebrovascular stroke, major adverse cardiovascular events (MACE), objective functional endurance, patient-reported health-related quality of life, alongside changes in anthropometric indices, systemic blood pressure, and circulating metabolic biomarkers. Across these included review artifacts, the aggregate patient enrollment spanned an expansive volume, ranging from a base cohort of 4,043 individuals to a maximum of 85,373 participants.

### Methodological quality assessment

Overall methodological quality was limited (23–33, 35). AMSTAR 2 classified six reviews as having low overall methodological confidence and five as having critically low overall methodological confidence; no review achieved high or moderate overall methodological confidence (Figure 2; Supplementary Table S1).

**Figure 2:**
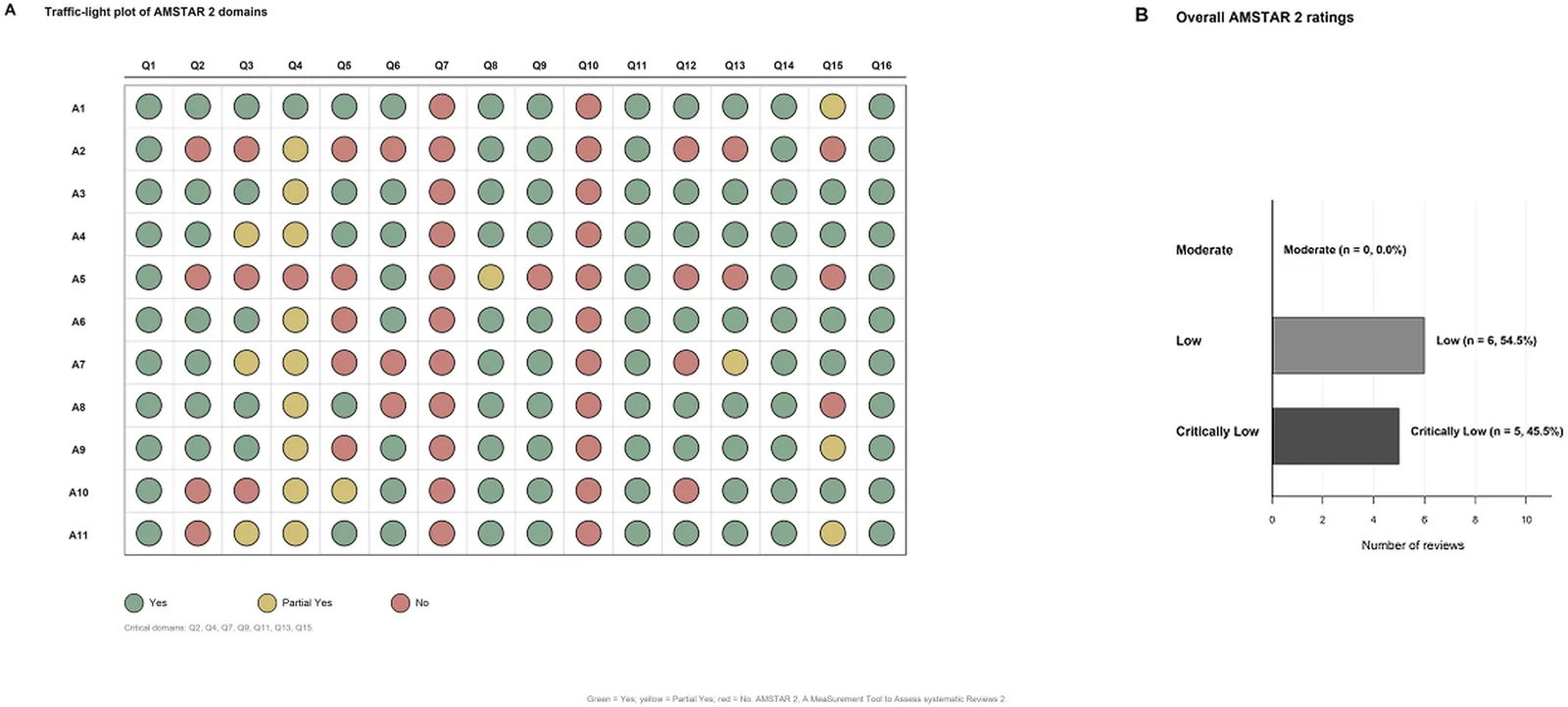
AMSTAR 2 methodological quality assessment of the 11 reviews contributing to the core review-level synthesis. Panel A shows the traffic-light plot across Q1 to Q16. Panel B shows the distribution of overall AMSTAR 2 methodological confidence ratings.

Several methodological strengths were common across the included reviews, including clearly framed questions, generally appropriate quantitative synthesis methods used by the included reviews, exploration of statistical heterogeneity, disclosure of conflicts of interest, risk-of-bias assessment, and reporting of primary-study characteristics. The most frequent weaknesses were failure to provide a list of excluded studies with reasons and failure to report funding sources for the included primary trials. Additional limitations in some reviews included incomplete searches, absence of independent duplicate study selection or data extraction, lack of prospective protocol registration, and limited or absent assessment of publication bias.

### Overlap analysis

The 11 reviews contributing to the core review-level synthesis relied substantially on shared primary evidence (37). The citation matrix contained 86 primary-study occurrences (N), representing 37 unique primary studies (c) across 11 reviews (r). The CCA was 13.2% (0.1324), which indicates high overlap according to established thresholds. The citation matrix and pairwise overlap matrix are presented in Supplementary Table S2. Overlap was concentrated among reviews specifically addressing obesity-related HFpEF and GLP-1-based interventions, whereas broader or outcome-specific reviews drew on a more dispersed evidence base. Because primary trials were used only for this overlap assessment, no trial-level outcome was double counted in the umbrella synthesis.

### Cardiovascular outcomes

Across the included evidence syntheses, the clearest directional agreement concerned heart failure-related outcomes (Table 2). For heart failure hospitalization, the review by Diallo et al. reported HR 0.78 (95% CI 0.73–0.84; *n* = 25,440). For the composite of cardiovascular death and heart failure hospitalization, Diallo et al. reported HR 0.84 (95% CI 0.78–0.89), Ahmed HM et al. reported HR 0.76 (95% CI 0.64–0.90), and Ahmed M et al. reported RR 0.73 (95% CI 0.57–0.93) (23, 24, 31). Although the magnitude and precision of the review-reported estimates varied, their direction was generally consistent. This agreement supports a potential benefit for heart failure decompensation, but it should not be interpreted as multiple fully independent confirmations because several reviews included the same primary trials.

**Table 2:** Summary of review-reported evidence on cardiovascular, functional, and safety outcomes of GLP-1-based therapies in obesity-related HFpEF.

Outcome	Direction of effect	Review-level interpretative classification	Participants (approx.)	Representative effect estimates reported by included reviews
Heart failure hospitalization	↓ Beneficial	Moderately consistent review-level evidence	25,000+	Diallo et al.: HR 0.78 (0.73–0.84)
Composite (CV death + HF hospitalization)	↓ Beneficial	Moderately consistent review-level evidence	70,000+	Diallo et al.: HR 0.84; Ahmed HM et al.: HR 0.76; Ahmed M et al.: RR 0.73
Cardiovascular mortality	Neutral/inconsistent	Limited consistency across reviews	variable	Ahmed HM et al., Cleto et al., and Diallo et al.: not consistently significant
All-cause mortality	Neutral/inconsistent	Limited consistency across reviews	variable	Ahmed HM et al., Cleto et al., and Diallo et al.: no consistent reduction
Major adverse cardiovascular events (MACE)	Possible benefit (uncertain)	Limited consistency across reviews	Limited	Gonzales-Uribe et al.: HR 0.71 (0.60–0.86)
Stroke	↓ Beneficial	Limited consistency across reviews	85,000+	Gera et al.: OR 0.85 (0.77–0.93)
Health-related quality of life (KCCQ)	↑ Beneficial	Moderately consistent review-level evidence	∼1,400	Chen et al.: +7 point improvement
Exercise capacity (6MWD)	↑ Beneficial	Moderately consistent review-level evidence	∼1,400	Chen et al.: +16–18 meters

In contrast, the reviews by Ahmed HM et al., Cleto et al., and Diallo et al. reported less consistent estimates for cardiovascular mortality across populations and analytical frameworks (23, 29, 31). Their findings for all-cause mortality were similarly discordant, precluding a conclusion that GLP-1-based therapy provides a uniform survival benefit in HFpEF.

For cerebrovascular outcomes, the review by Gera et al. reported OR 0.85 (95% CI 0.77–0.93; *n* = 85,373) for stroke, while Cleto et al. also reported estimates favoring a lower stroke risk across semaglutide-focused analyses (29, 32). However, heterogeneity of the underlying populations and the influence of large individual trials limited direct applicability to obesity-related HFpEF. For MACE, the review by Gonzales-Uribe et al. reported HR 0.71 (95% CI 0.60–0.86), although evidence was derived from fewer reviews and remained limited (33). Taken together, estimates for stroke, MACE, and mortality were derived primarily from broader cardiometabolic or mixed-HF populations and should be interpreted as supportive context rather than direct evidence for obesity-related HFpEF. Review-level findings were more consistent for heart failure-related outcomes than for mortality or other macrovascular endpoints; this is an umbrella-review interpretative classification, not a formal GRADE rating.

### Functional and quality-of-life outcomes

Included reviews evaluating obesity-related HFpEF generally reported improvements in health-related quality of life and exercise capacity. The review by Chen et al. reported representative improvements of approximately 7 points in Kansas City Cardiomyopathy Questionnaire (KCCQ) scores and approximately 16–18 m in 6-minute walk distance; findings in the same direction were reported by Ahmed HM et al., Caldeira Gaelzer et al., and Costa et al. (23, 26, 28, 30). The direction of these findings was consistent, but the similarity of estimates must be interpreted in light of partial overlap in the HFpEF trials included across reviews.

Taken together, the review-level evidence supports a consistent functional signal in obesity-related HFpEF, encompassing both patient-reported health status and objective exercise capacity. Functional outcomes were therefore classified as moderately consistent review-level evidence after considering methodological confidence, consistency, precision, sample size, clinical relevance, and overlap; this is an umbrella-review interpretative classification rather than a formal GRADE rating.

## Discussion

### Reappraisal of the research question and main findings

This umbrella review examined whether existing evidence syntheses support benefits of GLP-1-based therapies in obesity-related HFpEF beyond established metabolic effects (10–17, 44–46). Its principal contribution is a credibility assessment of the review literature, rather than a new estimate of treatment efficacy, combining AMSTAR 2 appraisal with formal quantification of review overlap.

Across included reviews, heart failure hospitalization and composite heart failure outcomes were reported in a generally favorable direction (23, 24, 28, 30, 31, 33), whereas mortality estimates were inconsistent or neutral (23, 25, 29–31, 33). Reviews of functional outcomes, including quality of life and exercise capacity, showed the most stable favorable direction (23, 26, 28, 30). These patterns describe the conclusions of existing reviews and do not represent effect estimates generated by this umbrella review.

This is not simply a contrast between positive and neutral outcomes. Rather, the review-level pattern suggests a separation between symptom- and hospitalization-related outcomes and survival outcomes in obesity-related HFpEF. GLP-1-based therapies may therefore primarily influence the metabolic-functional expression of the disease, while evidence for modification of long-term mortality remains insufficient.

### Interpretation of the main findings

At a mechanistic level, HFpEF is increasingly conceptualized as a systemic cardiometabolic syndrome rather than a purely cardiac disorder, in which metabolic dysfunction, inflammation, and peripheral limitations play central roles in symptom generation (4–7, 47–50). Within this framework, the observed pattern is biologically coherent: GLP-1-based therapies act predominantly on weight reduction, insulin resistance, and systemic metabolic stress, which directly influence exercise tolerance and congestion-related symptoms.

The generally favorable direction of review-reported heart failure hospitalization estimates (23, 24, 28, 30, 31, 33) may reflect improvements in volume sensitivity and metabolic efficiency rather than structural cardiac remodeling. However, the absence of a consistent mortality signal suggests that determinants of survival, including multimorbidity, renal dysfunction, and frailty, may extend beyond the principal biological pathways targeted by GLP-1-based therapies.

This interpretation also reframes the meaning of “efficacy” in HFpEF. Rather than representing incomplete translation of therapy, the divergence between functional and mortality outcomes may reflect a fundamental separation between reversible metabolic-peripheral determinants and irreversible systemic risk determinants.

### Positioning within existing evidence

The review-level pattern is broadly concordant with major randomized trials. STEP-HFpEF and STEP-HFpEF DM reported improvements in symptoms and functional capacity without establishing mortality benefit (19, 20). SELECT and FLOW reported cardiovascular and cardiorenal benefits in broader cardiometabolic populations, although their relevance to HFpEF-specific survival remains indirect (18, 22). SUMMIT further supported metabolic and functional benefits while leaving long-term prognostic effects uncertain (21).

The present umbrella review does not independently confirm these trial effects through re-analysis. Instead, it shows that the reviews synthesizing these trials generally converge on functional and heart failure-event benefits while remaining uncertain about mortality. The credibility of that convergence depends on review methods and on whether the contributing reviews are genuinely independent.

The CCA of 13.2% indicates high overlap among the included reviews. Thus, consistency across publications may partly reflect repeated analyses of shared landmark trials rather than replication across independent evidence bases. We therefore interpreted agreement using four complementary considerations: direction of findings, methodological quality, clinical relevance to obesity-related HFpEF, and the extent of overlap. Primary trials were used only to construct the overlap matrix and calculate the CCA; their outcome data were not re-counted or quantitatively recombined, so no trial-level double counting occurred in this umbrella review.

### Clinical interpretation and implications

Contemporary guideline-directed HFpEF management remains phenotype- and comorbidity-based. Sodium-glucose cotransporter-2 (SGLT2) inhibitors are recommended to reduce heart failure events across the preserved-ejection-fraction spectrum, and diuretics remain central for relief of congestion. Angiotensin receptor-neprilysin inhibitors and mineralocorticoid receptor antagonists may be considered in selected patients when blood pressure, renal function, serum potassium, ejection fraction, and the clinical phenotype are appropriate (1, 2, 39–42).

Nonpharmacological care is also integral to HFpEF management, including regular physical activity or structured exercise, dietary and lifestyle intervention, intentional weight management, and treatment of hypertension, atrial fibrillation, coronary disease, chronic kidney disease, sleep-disordered breathing, and diabetes. In obesity-related HFpEF, reducing adiposity and improving cardiometabolic health are phenotype-specific goals, but these measures should be integrated with established heart failure care (1, 2, 47, 49, 50).

Within this framework, GLP-1 receptor agonists and related incretin therapies should currently be viewed as adjunctive phenotype-directed therapies for patients with obesity and/or diabetes. Their most consistently review-reported benefits concern symptom burden, functional capacity, weight-related disease expression, and heart failure hospitalization, but they should not replace SGLT2 inhibitors, diuretic management, or other guideline-directed therapies used in appropriate patients. The current review-level evidence does not establish these agents as universal mortality-modifying therapy across the heterogeneous HFpEF population, and their role in non-obese or metabolically less prominent phenotypes remains uncertain.

### Broader interpretation of HFpEF phenotype

The findings reinforce the heterogeneity of HFpEF and the importance of phenotype-specific interpretation (4–7, 47–50). A relatively favorable functional response in obesity-related HFpEF is biologically consistent with a metabolically dominant phenotype. Nevertheless, several included reviews also incorporated mixed heart failure phenotypes, general cardiometabolic populations, and cohorts defined by overweight or diabetes rather than confirmed obesity-related HFpEF.

Evidence from these broader populations can support biological plausibility and provide contextual information, but it is not definitive evidence for obesity-related HFpEF. Estimates derived from mixed populations should therefore be interpreted according to their population fidelity and should not be generalized automatically to a narrowly defined HFpEF phenotype. The evidence-context classification in Table 1 further emphasizes that cardiometabolic outcome trials provide biological and clinical context but should not be interpreted as direct confirmation of treatment effects in obesity-related HFpEF. Accordingly, signals for stroke, MACE, and mortality, which arose mainly from broader cardiometabolic or mixed-HF populations, should be interpreted as supportive and not as direct evidence of treatment effects in obesity-related HFpEF.

This phenotype-dependent interpretation may explain why functional improvements appear more consistent than mortality effects. Patients with obesity-driven HFpEF may be especially responsive to metabolic interventions, whereas survival across HFpEF phenotypes is influenced by heterogeneous non-metabolic determinants that may not be directly modified by GLP-1 signaling.

### Limitations and interpretation constraints

Several limitations require emphasis. First, the CCA of 13.2% indicates high cross-review overlap in primary trials (37). Apparent consistency across reviews may therefore partly reflect repeated analyses of the same landmark trials rather than independent replication. We addressed this by interpreting outcomes according to direction, AMSTAR 2 methodological confidence, clinical relevance, and overlap, and by avoiding quantitative recombination or double counting of primary-trial outcomes. Second, AMSTAR 2 rated all core reviews as having low or critically low overall methodological confidence (35), which limits the robustness of the available synthesis-level evidence.

Third, several included reviews combined mixed heart failure phenotypes, broader cardiometabolic populations, or cohorts selected for overweight or diabetes. Findings from these populations are supportive but not definitive for obesity-related HFpEF, and phenotype-specific inferences remain limited. Fourth, mortality analyses in HFpEF-specific subgroups were often underpowered and had follow-up durations that may have been insufficient to detect long-term prognostic differences. Finally, this umbrella review was not prospectively registered in PROSPERO. The absence of prospective protocol registration may have reduced methodological transparency and introduced the possibility of *post hoc* methodological decisions, although predefined eligibility criteria and standardized umbrella-review methodology were applied consistently throughout the review.

### Future directions

Future studies should prioritize adequately powered, HFpEF-specific randomized trials with longer follow-up to determine whether early functional improvement translates into durable reductions in clinical events or mortality. Phenotype-stratified designs are needed to distinguish obesity-related HFpEF from other HFpEF subtypes and to assess comparative or complementary effects of GLP-1 receptor agonists, dual incretin co-agonists, SGLT2 inhibitors, and other guideline-directed therapies. Future systematic reviews should use prospective protocols, complete search strategies, transparent exclusion lists, and explicit management of overlapping trials.

## Conclusion

Existing systematic reviews and meta-analyses generally suggest that GLP-1-based therapies are associated with improved functional status and fewer heart failure hospitalizations in obesity-related HFpEF, while evidence for mortality benefit remains inconsistent. This umbrella review does not generate a new efficacy estimate; it evaluates the credibility of those published syntheses. Interpretation is constrained by low methodological quality, high review overlap, and inclusion of broader cardiometabolic populations. GLP-1-based therapies should therefore be regarded as emerging phenotype-directed metabolic-functional treatments, complementary to established HFpEF care, rather than universal mortality-modifying therapy.

## Data Availability

The original contributions presented in the study are included in the article/Supplementary Material, further inquiries can be directed to the corresponding author.
