# Supplementary material for: GLP-1-Based therapies for obesity-related heart failure with preserved ejection fraction: an umbrella review incorporating broader heart failure and cardiometabolic evidence
Source: Front Cardiovasc Med. 2026 Sep 30;13:1930278. doi: 10.3389/fcvm.2026.1930278 (PMC13661543; doi:10.3389/fcvm.2026.1930278)
Supplement: Supplementary file 1 [file Table1.docx]

**Supplementary Table S1.**

**AMSTAR 2 methodological quality assessment of included systematic reviews and meta-analyses**

| **Included review** | **D1** | **D2** | **D3** | **D4** | **D5** | **D6** | **D7** | **D8** | **D9** | **D10** | **D11** | **D12** | **D13** | **D14** | **D15** | **Overall confidence** |
| --- | --- | --- | --- | --- | --- | --- | --- | --- | --- | --- | --- | --- | --- | --- | --- | --- |
| **Ahmed HM, 2026** | Yes | Yes | No | Yes | Yes | Yes | No | Yes | Yes | No | Yes | Yes | Yes | Partial Yes | Yes | **Low** |
| **Ahmed M, 2025** | No | Partial Yes | No | Partial Yes | No | No | No | Yes | Yes | No | Yes | No | No | No | Yes | **Critically low** |
| **Behers, 2026** | Yes | Partial Yes | No | Partial Yes | Yes | Yes | No | Yes | Yes | No | Yes | Yes | Yes | Yes | Yes | **Low** |
| **Caldeira Gaelzer, 2026** | Yes | Partial Yes | No | Partial Yes | Yes | Yes | No | Yes | Yes | No | Yes | Yes | Yes | Yes | Yes | **Low** |
| **Patoulias, 2022** | No | No | No | No | No | Yes | No | Partial Yes | No | No | Yes | No | No | No | Yes | **Critically low** |
| **Chen, 2026** | Yes | Partial Yes | No | Partial Yes | No | Yes | No | Yes | Yes | No | Yes | Yes | Yes | Yes | Yes | **Low** |
| **Cleto, 2025** | Yes | Partial Yes | No | Partial Yes | No | No | No | Yes | Yes | No | Yes | No | Partial Yes | Yes | Yes | **Low** |
| **Costa, 2025** | Yes | Partial Yes | No | Partial Yes | Yes | No | No | Yes | Yes | No | Yes | Yes | Yes | No | Yes | **Critically low** |
| **Diallo, 2025** | Yes | Partial Yes | No | Partial Yes | No | Yes | No | Yes | Yes | No | Yes | Yes | Yes | Partial Yes | Yes | **Low** |
| **Gera, 2025** | No | Partial Yes | No | Partial Yes | Partial Yes | Yes | No | Yes | Yes | No | Yes | No | Yes | Yes | Yes | **Critically low** |
| **Gonzales-Uribe, 2025** | No | Partial Yes | No | Partial Yes | Yes | Yes | No | Yes | Yes | No | Yes | Yes | Yes | Partial Yes | Yes | **Critically low** |

Domain key: D1, Protocol registration; D2, Search strategy comprehensiveness; D3, Justification of excluded studies; D4, Literature search; D5, Duplicate study selection; D6, Duplicate data extraction; D7, List of excluded studies; D8, Included study characteristics; D9, Risk of bias assessment; D10, Sources of funding; D11, Appropriate meta-analysis methods; D12, Risk of bias consideration; D13, Interpretation of findings; D14, Publication bias assessment; D15, Conflicts of interest.

Ratings: Yes, Partial Yes, No, or Not applicable. Overall confidence: High, Moderate, Low, or Critically low. The source judgments were retained without rescoring. In this condensed submission layout, D2 and D4 retain the same source literature-search judgment, and D3 and D7 retain the same source excluded-study judgment.

Overall distribution: Low confidence, 6 reviews; Critically low confidence, 5 reviews; High or Moderate confidence, 0 reviews. AMSTAR 2, A MeaSurement Tool to Assess systematic Reviews 2.
