# Supplementary material for: GLP-1-Based therapies for obesity-related heart failure with preserved ejection fraction: an umbrella review incorporating broader heart failure and cardiometabolic evidence
Source: Front Cardiovasc Med. 2026 Sep 30;13:1930278. doi: 10.3389/fcvm.2026.1930278 (PMC13661543; doi:10.3389/fcvm.2026.1930278)
Supplement: Supplementary file 2 [file Table2.docx]

**Supplementary Table S2.**

**Citation matrix of overlapping primary studies among included reviews**

**CCA summary**

| **Metric** | **Symbol** | **Value** |
| --- | --- | --- |
| Number of included reviews | r | **11** |
| Number of primary-study occurrences | N | **86** |
| Number of unique primary studies | c | **37** |
| Corrected covered area | CCA | **13.2%** |
| Interpretation | — | **High overlap** |

For this table, r is the number of included reviews, N is the total number of primary-study occurrences, and c is the number of unique primary studies. CCA = (N - c) / (r × c - c) = 13.2%. The calculation assesses overlap only; primary-trial outcome data were not re-counted or pooled in the umbrella review.

**Panel A. Citation matrix, primary studies T1-T19**

| **Included review** | **T1** | **T2** | **T3** | **T4** | **T5** | **T6** | **T7** | **T8** | **T9** | **T10** | **T11** | **T12** | **T13** | **T14** | **T15** | **T16** | **T17** | **T18** | **T19** | **Total** |
| --- | --- | --- | --- | --- | --- | --- | --- | --- | --- | --- | --- | --- | --- | --- | --- | --- | --- | --- | --- | --- |
| **Ahmed HM, 2026** | **1** | **1** | **1** | **1** | **1** | **1** | **1** | **1** | 0 | 0 | 0 | 0 | 0 | 0 | 0 | 0 | 0 | 0 | 0 | **8** |
| **Ahmed M, 2025** | 0 | 0 | **1** | **1** | **1** | **1** | **1** | **1** | 0 | 0 | 0 | 0 | 0 | 0 | 0 | 0 | 0 | 0 | 0 | **6** |
| **Behers, 2026** | 0 | 0 | **1** | **1** | **1** | **1** | **1** | **1** | 0 | 0 | 0 | 0 | 0 | 0 | 0 | 0 | 0 | 0 | 0 | **6** |
| **Caldeira Gaelzer, 2026** | 0 | 0 | 0 | 0 | **1** | **1** | **1** | **1** | **1** | 0 | 0 | 0 | 0 | 0 | 0 | 0 | 0 | 0 | 0 | **5** |
| **Patoulias, 2022** | 0 | 0 | 0 | 0 | 0 | 0 | 0 | 0 | 0 | **1** | **1** | **1** | **1** | 0 | 0 | 0 | 0 | 0 | 0 | **4** |
| **Chen, 2026** | 0 | 0 | 0 | 0 | 0 | **1** | **1** | **1** | 0 | 0 | 0 | 0 | 0 | 0 | 0 | 0 | 0 | 0 | 0 | **3** |
| **Cleto, 2025** | 0 | 0 | 0 | 0 | **1** | **1** | **1** | 0 | 0 | **1** | 0 | 0 | **1** | **1** | **1** | **1** | **1** | **1** | **1** | **11** |
| **Costa, 2025** | 0 | 0 | **1** | **1** | **1** | **1** | **1** | **1** | 0 | 0 | 0 | 0 | 0 | 0 | 0 | 0 | 0 | 0 | 0 | **6** |
| **Diallo, 2025** | 0 | 0 | 0 | **1** | **1** | **1** | **1** | 0 | 0 | 0 | 0 | 0 | 0 | 0 | 0 | 0 | 0 | 0 | 0 | **4** |
| **Gera, 2025** | 0 | 0 | **1** | **1** | **1** | 0 | 0 | 0 | 0 | 0 | 0 | 0 | 0 | **1** | 0 | 0 | 0 | 0 | 0 | **4** |
| **Gonzales-Uribe, 2025** | 0 | 0 | 0 | **1** | **1** | **1** | **1** | **1** | **1** | 0 | 0 | 0 | 0 | 0 | 0 | 0 | 0 | 0 | 0 | **6** |

Cells: 1 = primary study included in the review; 0 = primary study not included.

**Panel B. Citation matrix, primary studies T20-T37**

| **Included review** | **T20** | **T21** | **T22** | **T23** | **T24** | **T25** | **T26** | **T27** | **T28** | **T29** | **T30** | **T31** | **T32** | **T33** | **T34** | **T35** | **T36** | **T37** | **Total** |
| --- | --- | --- | --- | --- | --- | --- | --- | --- | --- | --- | --- | --- | --- | --- | --- | --- | --- | --- | --- |
| **Ahmed HM, 2026** | 0 | 0 | 0 | 0 | 0 | 0 | 0 | 0 | 0 | 0 | 0 | 0 | 0 | 0 | 0 | 0 | 0 | 0 | **0** |
| **Ahmed M, 2025** | 0 | 0 | 0 | 0 | 0 | 0 | 0 | 0 | 0 | 0 | 0 | 0 | 0 | 0 | 0 | 0 | 0 | 0 | **0** |
| **Behers, 2026** | 0 | 0 | 0 | 0 | 0 | 0 | 0 | 0 | 0 | 0 | 0 | 0 | 0 | 0 | 0 | 0 | 0 | 0 | **0** |
| **Caldeira Gaelzer, 2026** | 0 | 0 | 0 | 0 | 0 | 0 | 0 | 0 | 0 | 0 | 0 | 0 | 0 | 0 | 0 | 0 | 0 | 0 | **0** |
| **Patoulias, 2022** | 0 | 0 | 0 | 0 | 0 | 0 | 0 | 0 | 0 | 0 | 0 | 0 | 0 | 0 | 0 | 0 | 0 | 0 | **0** |
| **Chen, 2026** | 0 | 0 | 0 | 0 | 0 | 0 | 0 | 0 | 0 | 0 | 0 | 0 | **1** | **1** | **1** | **1** | **1** | **1** | **6** |
| **Cleto, 2025** | **1** | **1** | **1** | **1** | **1** | **1** | 0 | 0 | 0 | 0 | 0 | 0 | 0 | 0 | 0 | 0 | 0 | 0 | **6** |
| **Costa, 2025** | 0 | 0 | 0 | 0 | 0 | 0 | 0 | 0 | 0 | 0 | 0 | 0 | 0 | 0 | 0 | 0 | 0 | 0 | **0** |
| **Diallo, 2025** | 0 | 0 | 0 | 0 | 0 | 0 | 0 | 0 | 0 | 0 | 0 | 0 | **1** | **1** | **1** | **1** | 0 | 0 | **4** |
| **Gera, 2025** | **1** | 0 | 0 | 0 | 0 | 0 | **1** | **1** | **1** | **1** | **1** | **1** | 0 | 0 | 0 | 0 | 0 | 0 | **7** |
| **Gonzales-Uribe, 2025** | 0 | 0 | 0 | 0 | 0 | 0 | 0 | 0 | 0 | 0 | 0 | 0 | 0 | 0 | 0 | 0 | 0 | 0 | **0** |

Cells: 1 = primary study included in the review; 0 = primary study not included.

**Panel C. Pairwise number of shared primary studies**

| **Included review** | **Ahmed HM, 2026** | **Ahmed M, 2025** | **Behers, 2026** | **Caldeira Gaelzer, 2026** | **Patoulias, 2022** | **Chen, 2026** | **Cleto, 2025** | **Costa, 2025** | **Diallo, 2025** | **Gera, 2025** | **Gonzales-Uribe, 2025** |
| --- | --- | --- | --- | --- | --- | --- | --- | --- | --- | --- | --- |
| **Ahmed HM, 2026** | **8** | 6 | 6 | 4 | 0 | 3 | 3 | 6 | 4 | 3 | 5 |
| **Ahmed M, 2025** | 6 | **6** | 6 | 4 | 0 | 3 | 3 | 6 | 4 | 3 | 5 |
| **Behers, 2026** | 6 | 6 | **6** | 4 | 0 | 3 | 3 | 6 | 4 | 3 | 5 |
| **Caldeira Gaelzer, 2026** | 4 | 4 | 4 | **5** | 0 | 3 | 3 | 4 | 3 | 1 | 5 |
| **Patoulias, 2022** | 0 | 0 | 0 | 0 | **4** | 0 | 2 | 0 | 0 | 0 | 0 |
| **Chen, 2026** | 3 | 3 | 3 | 3 | 0 | **9** | 2 | 3 | 6 | 0 | 3 |
| **Cleto, 2025** | 3 | 3 | 3 | 3 | 2 | 2 | **17** | 3 | 3 | 3 | 3 |
| **Costa, 2025** | 6 | 6 | 6 | 4 | 0 | 3 | 3 | **6** | 4 | 3 | 5 |
| **Diallo, 2025** | 4 | 4 | 4 | 3 | 0 | 6 | 3 | 4 | **8** | 2 | 4 |
| **Gera, 2025** | 3 | 3 | 3 | 1 | 0 | 0 | 3 | 3 | 2 | **11** | 2 |
| **Gonzales-Uribe, 2025** | 5 | 5 | 5 | 5 | 0 | 3 | 3 | 5 | 4 | 2 | **6** |

Diagonal cells show the number of distinct primary studies identified within each review; off-diagonal cells show the number shared by each pair of reviews.

**Primary-study key**

| **ID** | **Primary study/trial** | **First author** | **Year** | **Design** | **Population** | **Intervention** |
| --- | --- | --- | --- | --- | --- | --- |
| **T1** | **FIGHT** | Margulies | 2016 | RCT | HFrEF | Liraglutide |
| **T2** | **LIVE** | Jorsal | 2017 | RCT | HFrEF | Liraglutide |
| **T3** | **EXSCEL** | Holman | 2017 | RCT/post-hoc HF analysis | Type 2 diabetes with cardiovascular risk; HF subgroup where reported | Exenatide |
| **T4** | **FLOW** | Pratley | 2024 | RCT/post-hoc HF analysis | Type 2 diabetes and chronic kidney disease; HF subgroup where reported | Semaglutide |
| **T5** | **SELECT** | Lincoff | 2023 | RCT/prespecified HF analysis | Overweight or obesity without diabetes; prevalent HF subgroup where reported | Semaglutide |
| **T6** | **STEP-HFpEF** | Kosiborod | 2023 | RCT | Obesity-related HFpEF | Semaglutide |
| **T7** | **STEP-HFpEF DM** | Kosiborod | 2024 | RCT | Obesity-related HFpEF with type 2 diabetes | Semaglutide |
| **T8** | **SUMMIT** | Packer | 2025 | RCT | HFpEF and obesity | Tirzepatide |
| **T9** | **Wallner registry cohort** | Wallner | 2024 | Retrospective cohort | Heart failure and type 2 diabetes in Swedish registries | GLP-1 receptor agonists |
| **T10** | **STEP 1** | Wilding | 2021 | RCT | Adults with overweight or obesity without diabetes | Semaglutide |
| **T11** | **STEP 2** | Davies | 2021 | RCT | Adults with overweight or obesity and type 2 diabetes | Semaglutide |
| **T12** | **STEP 3** | Wadden | 2021 | RCT | Adults with overweight or obesity receiving intensive behavioral therapy | Semaglutide |
| **T13** | **STEP 4** | Rubino | 2021 | RCT | Adults with overweight or obesity after semaglutide run-in | Semaglutide |
| **T14** | **SUSTAIN-6** | Marso | 2016 | RCT | Type 2 diabetes at high cardiovascular risk | Semaglutide |
| **T15** | **Dwibedi 2024 semaglutide-dapagliflozin trial** | Dwibedi | 2024 | Randomized open-label trial | Type 2 diabetes of different pathophysiology | Semaglutide and dapagliflozin |
| **T16** | **STEP TEENS** | Weghuber | 2022 | RCT | Adolescents with obesity | Semaglutide |
| **T17** | **PIONEER 5** | Mosenzon | 2019 | RCT | Type 2 diabetes with moderate renal impairment | Oral semaglutide |
| **T18** | **Sivalingam 2024 semaglutide add-on trial** | Sivalingam | 2024 | RCT | Type 2 diabetes on empagliflozin with albuminuria | Semaglutide added to empagliflozin |
| **T19** | **PIONEER 1** | Aroda | 2019 | RCT | Type 2 diabetes | Oral semaglutide |
| **T20** | **PIONEER 6** | Husain | 2019 | RCT | Type 2 diabetes at high cardiovascular risk | Oral semaglutide |
| **T21** | **PIONEER 9** | Yamada | 2020 | RCT | Japanese patients with type 2 diabetes | Oral semaglutide |
| **T22** | **STEP 8** | Rubino | 2022 | RCT | Adults with overweight or obesity without diabetes | Semaglutide versus liraglutide |
| **T23** | **PIONEER 10** | Yabe | 2020 | RCT | Japanese patients with type 2 diabetes | Oral semaglutide versus dulaglutide |
| **T24** | **OASIS 1** | Knop | 2023 | RCT | Adults with overweight or obesity | Oral semaglutide 50 mg |
| **T25** | **PIONEER PLUS** | Aroda | 2023 | RCT | Adults with type 2 diabetes | Oral semaglutide 25 mg or 50 mg versus 14 mg |
| **T26** | **REWIND** | Gerstein | 2019 | RCT | Type 2 diabetes with or without previous cardiovascular disease | Dulaglutide |
| **T27** | **LEADER** | Marso | 2016 | RCT | Type 2 diabetes at high cardiovascular risk | Liraglutide |
| **T28** | **HARMONY Outcomes** | Hernandez | 2018 | RCT | Type 2 diabetes and cardiovascular disease | Albiglutide |
| **T29** | **ELIXA** | Pfeffer | 2015 | RCT | Type 2 diabetes after acute coronary syndrome | Lixisenatide |
| **T30** | **AMPLITUDE-O** | Gerstein | 2021 | RCT | Type 2 diabetes with cardiovascular or kidney disease | Efpeglenatide |
| **T31** | **FREEDOM-CVO** | Ruff | 2022 | RCT | Type 2 diabetes | Exenatide infusion |
| **T32** | **DELIVER** | Solomon | 2022 | RCT | HFmrEF or HFpEF | Dapagliflozin |
| **T33** | **EMPEROR-Preserved** | Anker | 2021 | RCT | HFpEF | Empagliflozin |
| **T34** | **TOPCAT** | Pitt | 2014 | RCT | HFpEF | Spironolactone |
| **T35** | **FINEARTS-HF** | Solomon | 2024 | RCT | HFmrEF or HFpEF | Finerenone |
| **T36** | **CHARM-Preserved** | Yusuf | 2003 | RCT | HFpEF | Candesartan |
| **T37** | **PARAGON-HF** | Solomon | 2019 | RCT | HFpEF | Sacubitril/valsartan |

Each standardized primary-study record appears once in the key and once as a matrix column, preventing duplicate counting of the same trial within the overlap assessment. Source limitation retained from the analysis file: A6 and A7 did not enumerate every included study name in the available PDF text; only clearly identifiable quantitative primary studies were entered.

CCA, corrected covered area; CV, cardiovascular; DM, diabetes mellitus; HF, heart failure; HFpEF, heart failure with preserved ejection fraction; HFrEF, heart failure with reduced ejection fraction; RCT, randomized controlled trial.
