# Supplementary material for: GLP-1-Based therapies for obesity-related heart failure with preserved ejection fraction: an umbrella review incorporating broader heart failure and cardiometabolic evidence
Source: Front Cardiovasc Med. 2026 Sep 30;13:1930278. doi: 10.3389/fcvm.2026.1930278 (PMC13661543; doi:10.3389/fcvm.2026.1930278)
Supplement: Supplementary file 3 [file Table3.docx]

**Supplementary Table S3. Database-specific search strategies**

| **Database** | **Database-specific search strategy** |
| --- | --- |
| **PubMed** | ((("Heart Failure, Diastolic"[MeSH Terms] OR "heart failure with preserved ejection fraction"[Title/Abstract] OR HFpEF[Title/Abstract] OR "heart failure with mildly reduced ejection fraction"[Title/Abstract] OR HFmrEF[Title/Abstract] OR obesity[MeSH Terms] OR obes*[Title/Abstract] OR "Metabolic Syndrome"[MeSH Terms] OR cardiometabolic[Title/Abstract]) AND ("Glucagon-Like Peptide 1"[MeSH Terms] OR "glucagon-like peptide-1 receptor agonist*"[Title/Abstract] OR "GLP-1 receptor agonist*"[Title/Abstract] OR semaglutide[Title/Abstract] OR liraglutide[Title/Abstract] OR exenatide[Title/Abstract] OR tirzepatide[Title/Abstract] OR "dual GLP-1/GIP"[Title/Abstract]) AND ("Systematic Review"[Publication Type] OR "Meta-Analysis"[Publication Type] OR "systematic review"[Title/Abstract] OR meta-analy*[Title/Abstract] OR "network meta-analysis"[Title/Abstract]))) AND ("2016/07/01"[Date - Publication] : "2026/06/30"[Date - Publication]) |
| **Embase** | ('heart failure with preserved ejection fraction'/exp OR 'heart failure with preserved ejection fraction':ti,ab,kw OR hfpEF:ti,ab,kw OR 'heart failure with mildly reduced ejection fraction':ti,ab,kw OR hfmREF:ti,ab,kw OR 'obesity'/exp OR obes*:ti,ab,kw OR 'metabolic syndrome'/exp OR cardiometabolic:ti,ab,kw) AND ('glucagon like peptide 1 receptor agonist'/exp OR 'glucagon-like peptide-1 receptor agonist*':ti,ab,kw OR 'GLP-1 receptor agonist*':ti,ab,kw OR semaglutide:ti,ab,kw OR liraglutide:ti,ab,kw OR exenatide:ti,ab,kw OR tirzepatide:ti,ab,kw OR 'dual GLP-1/GIP':ti,ab,kw) AND ('systematic review'/de OR 'meta analysis'/de OR 'systematic review':ti,ab,kw OR meta-analy*:ti,ab,kw OR 'network meta-analysis':ti,ab,kw) AND [2016-2026]/py. Results were limited to records published from 1 July 2016 through 30 June 2026. |
| **Web of Science** | TS=(("heart failure with preserved ejection fraction" OR HFpEF OR "heart failure with mildly reduced ejection fraction" OR HFmrEF OR obes* OR "metabolic syndrome" OR cardiometabolic) AND ("glucagon-like peptide-1 receptor agonist*" OR "GLP-1 receptor agonist*" OR semaglutide OR liraglutide OR exenatide OR tirzepatide OR "dual GLP-1/GIP") AND ("systematic review" OR meta-analy* OR "network meta-analysis")) AND PY=(2016-2026). Results were limited to records published from 1 July 2016 through 30 June 2026. |
| **Cochrane Library** | #1 MeSH descriptor: [Heart Failure, Diastolic] explode all trees; #2 ("heart failure with preserved ejection fraction" OR HFpEF OR "heart failure with mildly reduced ejection fraction" OR HFmrEF):ti,ab,kw; #3 MeSH descriptor: [Obesity] explode all trees; #4 MeSH descriptor: [Metabolic Syndrome] explode all trees; #5 (obes* OR cardiometabolic):ti,ab,kw; #6 MeSH descriptor: [Glucagon-Like Peptide 1] explode all trees; #7 ("glucagon-like peptide-1 receptor agonist*" OR "GLP-1 receptor agonist*" OR semaglutide OR liraglutide OR exenatide OR tirzepatide OR "dual GLP-1/GIP"):ti,ab,kw; #8 ("systematic review" OR meta-analy* OR "network meta-analysis"):ti,ab,kw; #9 (#1 OR #2 OR #3 OR #4 OR #5) AND (#6 OR #7) AND #8. Results were limited to records published from 1 July 2016 through 30 June 2026. |

*Search concepts were adapted to each database and combined terms for HFpEF/HFmrEF or obesity-related cardiometabolic populations, GLP-1-based therapies, and secondary evidence-synthesis designs. The search period was 1 July 2016 to 30 June 2026.*
